# My Baby’s Movements: a stepped wedge cluster randomised controlled trial to raise maternal awareness of fetal movements during pregnancy study protocol

**DOI:** 10.1186/s12884-019-2575-1

**Published:** 2019-11-21

**Authors:** V. Flenady, G. Gardener, F. M. Boyle, E. Callander, M. Coory, C. East, D. Ellwood, A. Gordon, K. M. Groom, P. F. Middleton, J. E. Norman, K. A. Warrilow, M. Weller, A. M. Wojcieszek, C. Crowther, on behalf of the IMPACT Clinical Trials Network for Mothers’ and Babies’ Health

**Affiliations:** 1 Centre of Research Excellence in Stillbirth, Mater Research Institute, The University of Queensland, Level 3 Aubigny Place Mater Research, South Brisbane QLD, Brisbane, 4101 Australia; 2 Department of Maternal Fetal Medicine, Mater Misericordiae Limited, Brisbane, Australia; 3 Institute for Social Science Research, The University of Queensland, Brisbane, Australia; 4 School of Medicine, Griffith University, Gold Coast, Australia; 5 School of Nursing and Midwifery, Monash University and Monash Women’s Maternity Services, Clayton, Victoria Australia; 6 School of Nursing & Midwifery, La Trobe University, Melbourne, Brazil; 7 Gold Coast University Hospital, Southport, Australia; 8 Sydney Medical School, University of Sydney, Sydney, Australia; 9 Liggins Institute, University of Auckland, Auckland, New Zealand; 10 SAHMRI Women and Kids, South Australian Health and Medical Research Institute, Adelaide, Australia; 11 Faculty of Health Sciences, University of Bristol, Bristol, UK

**Keywords:** Decreased fetal movements, Stillbirth, Best practice, Mobile phone application, Maternity care

## Abstract

**Background:**

Stillbirth is a devastating pregnancy outcome that has a profound and lasting impact on women and families. Globally, there are over 2.6 million stillbirths annually and progress in reducing these deaths has been slow. Maternal perception of decreased fetal movements (DFM) is strongly associated with stillbirth. However, maternal awareness of DFM and clinical management of women reporting DFM is often suboptimal. The My Baby’s Movements trial aims to evaluate an intervention package for maternity services including a mobile phone application for women and clinician education (MBM intervention) in reducing late gestation stillbirth rates.

**Methods/design:**

This is a stepped wedge cluster randomised controlled trial with sequential introduction of the MBM intervention to 8 groups of 3–5 hospitals at four-monthly intervals over 3 years.

The target population is women with a singleton pregnancy, without lethal fetal abnormality, attending for antenatal care and clinicians providing maternity care at 26 maternity services in Australia and New Zealand. The primary outcome is stillbirth from 28 weeks’ gestation. Secondary outcomes address: a) neonatal morbidity and mortality; b) maternal psychosocial outcomes and health-seeking behaviour; c) health services utilisation; d) women’s and clinicians’ knowledge of fetal movements; and e) cost. 256,700 births (average of 3170 per hospital) will detect a 30% reduction in stillbirth rates from 3/1000 births to 2/1000 births, assuming a significance level of 5%. Analysis will utilise generalised linear mixed models.

**Discussion:**

Maternal perception of DFM is a marker of an at-risk pregnancy and commonly precedes a stillbirth. MBM offers a simple, inexpensive resource to reduce the number of stillborn babies, and families suffering the distressing consequences of such a loss. This large pragmatic trial will provide evidence on benefits and potential harms of raising awareness of DFM using a mobile phone app.

**Trial registration:**

ACTRN12614000291684. Registered 19 March 2014.

**Version:**

Protocol Version 6.1, February 2018.

## Background

Stillbirth has profound and long-lasting adverse psychosocial and economic impacts on women and families, and also on health systems and society [1]. In 2015, an estimated 2.6 million stillbirths occurred globally with rates showing little or no decline [2]. While the majority of stillbirths occur in low and middle income countries, [2] high-income countries (HIC) still have substantial numbers of preventable stillbirths, [3] particularly beyond 28 weeks’ gestation, where survival for those born alive approaches 100% [4].

In HIC settings, stillbirth is now over twenty times more common than Sudden Unexpected Deaths in Infancy [5] where focussed prevention strategies, including awareness campaigns, have reduced these deaths by over 80% [6]. The scale of the hidden tragedy of stillbirth was the impetus for *The Lancet* to publish the 2011 [7–11] and 2016 stillbirths series [1–3, 12, 13] with a global call to action [12] to reduce preventable stillbirths focussing on births of 28 weeks’ or more gestation (late gestation). The Lancet’s 2016 series showed wide variation in late gestation stillbirth rates across 49 HIC ranging from 1.7/1000 to 8.8/1000 births, [3] highlighting the potential to further reduce stillbirth rates in such settings. In this series, New Zealand and Australia ranked 10th and 15th with rates of 2.3 and 2.7/1000 births respectively, indicating the potential for focussed attention to reduce preventable stillbirth [3].

In Australia and New Zealand, using the standard definition of stillbirth of 20 weeks or 400 g birthweight, the most recent annual data shows 2107 (6.7/1000) in Australia, [4] and 457 (7.5/1000) in New Zealand [14]; equating to seven deaths every day. Women who are socially disdavantaged have around twice the risk of stillbirth [3]. Ethnicity is also associated with stillbirth; Indigenous Australian women, [4] Pacific Islanders in New Zealand [14] and South Asian born women [15] have around twice the risk.

### The challenge of stillbirth prevention

With improvements in intrapartum care in HIC settings, the majority of stillbirths now occur in the antepartum period [8]. The on-going risk of stillbirth increases each week towards the end of pregnancy [16]. In addtition, maternal and fetal factors which increase the risk include maternal overweight and obesity, smoking in pregnancy, age 35 years or more, fetal growth restriction, and previous stillbirth [17]. In the absence of a reliable screening test for stillbirth, early identificaiton of risk factors combined with appropriate monitoring and early birth, when indicated, is the mainstay of management to reduce late gestation stillbirth [3].

DFM is a marker of an at-risk pregnancy [18, 19]. DFM is thought to be an adaptive response to acute or chronic placental dysfunction whereby the fetus reduces gross movement to conserve blood flow for the vital organs [19]. Women who experience DFM have a four-fold increased risk of stillbirth [19–21] and double the risk of fetal growth restriction [22, 23]. DFM is also associated with other serious adverse outcomes including feto-maternal haemorrhage, low birth weight, neonatal death and neurodevelopmental disability [24]. Clinical audits into substandard care show that around 20–30% of stillbirths may have been avoided through better care, [8] with deficiencies in care around detection and management of women with decreased/reduced fetal movements a common finding [25–27].

### DFM and the evidence for raising awareness

No universally agreed definition of DFM currently exists [28] and none are sufficiently robust as a screening tool for adverse pregnancy outcomes [29]. Maternal perception of DFM is more effective in detecting at-risk pregnancies than any threshold based on maternal count of fetal movements [18]. Many stillbirths are preceded by perceived DFM for a number of days [19, 30] and mothers who delay reporting DFM increases the risk of stillbirth [31].

A systematic revew of interventions to raise awareness and improve outcomes for women with DFM showed no clear beneift [32]. Fetal movement counting (where women record the number of movements using a kick chart) has been proposed as an intervention to reduce stillbirth rates through increasing maternal awareness of DFM. However, the Cochrane systematic review on fetal movement (FM) counting showed no reduction in stillbirths [33]. In the largest trial of kick counting, [34] while no reduction was shown in stillbirth rates, the overall late gestation stillbirth rate fell during the study period from 4 per 1000 to 2.8 per 1000 births. It was postulated that this reduction was due to an increased awareness and vigilance of DFM [28]. In a non-randomised quality improvement study across 14 hospitals in Norway [35, 36] a similar reduction was shown for a package of care to raise awareness of DFM (with optional kick counting) and a standardised protocol for clinical management. Importantly, in the Norwegian study women with DFM presented for care earlier during the intervention period. A more recent indiviual participant randomised controlled trial [37] showed that kick counting increased antenatal detection of FGR. While the trial was not designed to detect a difference in stillbirth rates, no difference was shown in the proportion of women presenting with DFM, induction rates, maternal concern about the baby [38] or maternal fetal attachment [22]. Recruitment into this trial was low (< 20% of all eligible women), suggesting that kick counting may not be widely acceptable to women. The recent AFFIRM trial in the UK [39] showed that a package of care to improve awareness and management of women with DFM resulted in an increase in induction of labour, caesarean section and neonatal admission to special care nursery. A non-statistically significant reduction in stillbirth rates of 10% was shown. The trial investigators concluded that changes in practice around DFM should await the results of ongoing trials.

Planned early birth to avoid stillbirth may result in increased maternal and neonatal adverse outcomes. While the risks of preterm birth are well understood, recent sudies have shown early-term birth (at 37 and 38 weeks) is associated with adverse neonatal and childhood outcomes [40]. Therefore, the risk of stillbirth must be carefully weighed against the risks associated with early birth at a given gestational age [41].

### Practices in Australia and New Zealand

A survey of obstetricians in Australia and New Zealand on care for women with DFM showed the majority agreed that maternal concern was the most valid definition of DFM [42]. However, low awareness and knowledge of the importance of DFM, and suboptimal practice in response to women’s reports of DFM, is evident [24]. Incorrect beliefs are common place, such as that a decrease in movements towards the end of pregnancy is normal [43]. Women often report not receiving information about DFM and many delay seeking care when DFM occurs [44]. Women who do receive information are more likely to know what to do when concerned [45]. To enhance consistency and quality of care for women presenting with DFM, a clinical practice guideline on DFM has been developed [24] with an accompanying brochure for women, which has been translated into 19 languages [46]. An e-learning program for clinicians has also been developed [47].

## Methods/design

### Aim

The aim of the MBM trial is to evaluate the effectiveness of a mobile phone application for women combined with an educational program for clinicians (MBM intervention) in reducing late gestation stillbirth rates.

### The My Baby’s Movements mobile phone program

mHealth is increasingly a feature of the healthcare landscape and mobile phone applications (apps) are widely used by pregnant women, who rate them as helpful for providing general reassurance and information about fetal development [48, 49]. Although the effects of mobile phone apps on pregnancy outcomes are yet to be established, [50] their reach and acceptability levels mean that this form of technology holds much promise for the delivery of appropriately tailored evidence-based information.

**Fig. 1:**
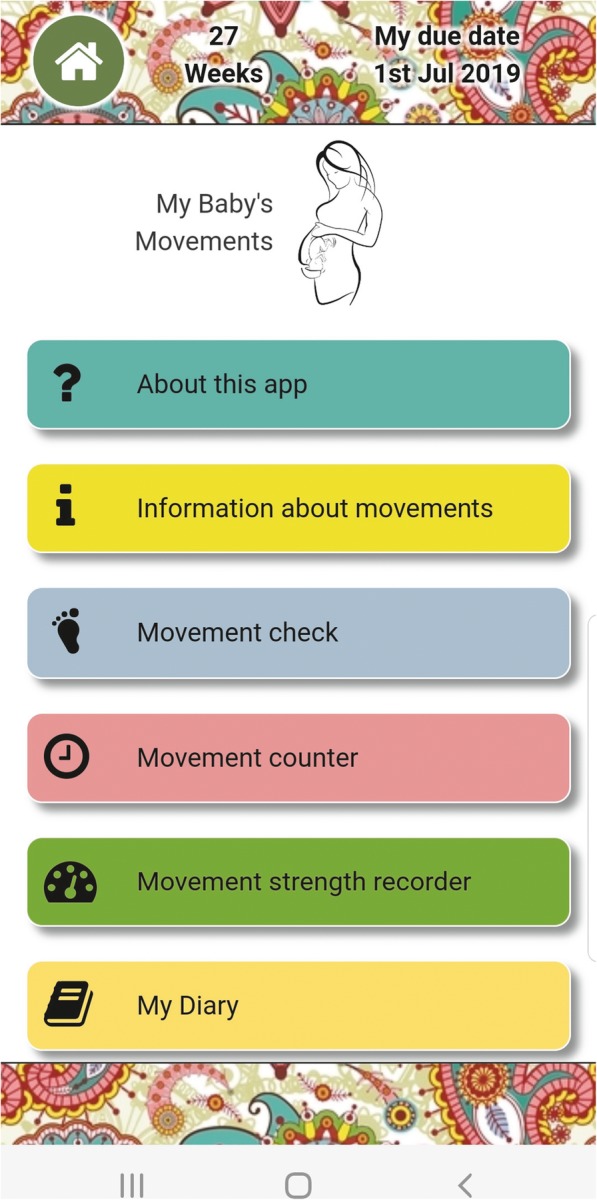
My Baby’s Movement application. Display of MBM app opening page

The My Baby’s Movement mobile phone program (MBM phone program) consists of a mobile phone app (Fig. 1), or a short message service (SMS) based program for women without access to a smartphone. The aim of the MBM phone program is to provide quality information about fetal movements to pregnant women, and to encourage early reporting if a decrease in movement occurs. A digital innovation company was contracted to create the MBM phone program to ensure an end-product that was accessible, engaging and interactive for users. Development included user engagement involving focus groups and interviews with pregnant women and a focus group with clinicians was conducted at the Mater Mothers’ Hospital (MMH). The MMH is a large tertiary service in Queensland, Australia and was chosen for convenience, [51] as it co-located with the main coordinating centre (Mater Research Institute, University of Queensland) and runs a specialised service for Aboriginal and Torres Strait Islander women, enabling ready access to potential participants for this work. Key considerations in development of the MBM program included acceptability and expectations of content and its delivery, cultural appropriateness, health literacy, and patient beliefs and misperceptions. All communications via the MBM app and SMS were designed to be delivered in a supportive and non-alarmist manner. Consultation with Aboriginal and Torres Strait Islander researchers, clinicians and community representatives provided essential feedback and ultimately a fetal movement information brochure tailored to Indigenous women and modifications to the MBM app including minor wording changes and a visual ‘theme’ option that incorporated Indigenous artwork.

The MBM app sends an alert to prompt the woman’s awareness of her baby’s movements at a time and frequency of her choice, from 28 week’s gestation until birth. If concerned, she is encouraged to contact her health care provider without delay. The app also provides a ‘movement counter’ option where the woman can record the number of movements she feels over a two-hour period, and a strength recorder option where she can record the strength of her baby’s movements if she wishes. This information is stored in the woman’s app diary for her review at any time. The alternative SMS program sends a series of messages to the woman on a weekly basis from 28 to 33 + 6 weeks’ gestation, and a twice-weekly basis from 34 to 42 weeks’ gestation. The messages include facts about fetal movements and a prompt to contact her healthcare provider if concerned about her baby’s movements. This is a one-way messaging system with no option for women to seek further advice or care via the MBM SMS. Accordingly, all SMS texts included the words ‘Do not reply’.

### Hypothesis

The primary hypothesis is that the MBM intervention will result in a reduction in stillbirth rates at 28 weeks’ or more gestation in women with a singleton pregnancy from 3/1000 to 2/1000. The baseline stillbirth rate is based on outcome data from the participating hospitals.

### Outcome measures

#### Primary endpoint

Stillbirth rates 28 weeks’ or more gestation in women with a singleton pregnancy.

#### Secondary endpoints are as follows

- Newborn outcomes: a composite measure of adverse outcome defined as one or more of the following - stillbirth, hypoxic ischemic encephalopathy, neonatal seizures; Meconium Aspiration Syndrome; stillbirths (20 weeks’ gestation or more); gestation at birth; birthweight; FGR at birth; Apgar Score < 7 at 5 min; umbilical artery pH < 7.0; intubation and ventilation at birth; use of mechanical ventilation; neonatal death (death of a live born infant regardless of gestation or birthweight); neonatal death at 28 weeks’ or more gestation.
- Obstetric outcomes: induction of labour; caesarean section, intrapartum and postpartum infection; postpartum haemorrhage; maternal admission to intensive care.
- Health service utilisation measures: episodes of women presenting with DFM at > 28 weeks’ gestation; antenatal admission to hospital for DFM; antenatal ultrasound; duration of neonatal intensive care, special care nursery and total hospital stay; and maternal length of hospital stay.
- Woman’s psychosocial outcomes and health seeking behaviour and acceptability: Maternal reporting of DFM delayed by > 24 h; acceptability of information on DFM and of MBM; women’s and clinicians’ knowledge of FM; maternal-fetal attachment (the Prenatal Attachment Inventory (PAI)) [52]; maternal pregnancy-related worries and concerns (the Cambridge Worry Scale Score [53];) anxiety (State-Trait Anxiety Index [54]); the Edinburgh Postnatal Depression Scale (EPDS) [55];; quality of life (QoL)(AQol8D) [56]; and health status (SF36, 57] at the end of pregnancy (or birth) at 6 months postpartum.

### Cost-effectiveness

A within trial cost-effectiveness analysis has also been designed to identify the incremental cost-effectiveness ratio of the mobile phone app and clinical education intervention relative to standard care. All costs to the health care system will be included, and the outcome of interest is the change in number of stillbirths.

### Patient and public involvement statement

The My Baby’s Movements trial is an endorsed trial of the Perinatal Society of Australia and New Zealand (PSANZ) Interdisciplinary Maternal and Perinatal Australasian Collaborative Trials (IMPACT) Network. An integral part of the development of IMPACT Network endorsed trials is consultation with consumers through consultation with the PSANZ Consumer Advisory Group and feedback at open forums. The voice of parents and the public was further incorporated into the MBM trial development through partnership with the Stillbirth Foundation Australia and through focus groups and one-to-one interviews as part of the MBM app development phase. Consultation with Aboriginal women was enabled through an Indigenous stillbirth advisory group established specifically for the MRI-UQ stillbirth research program.

### Study design

**Fig. 2:**
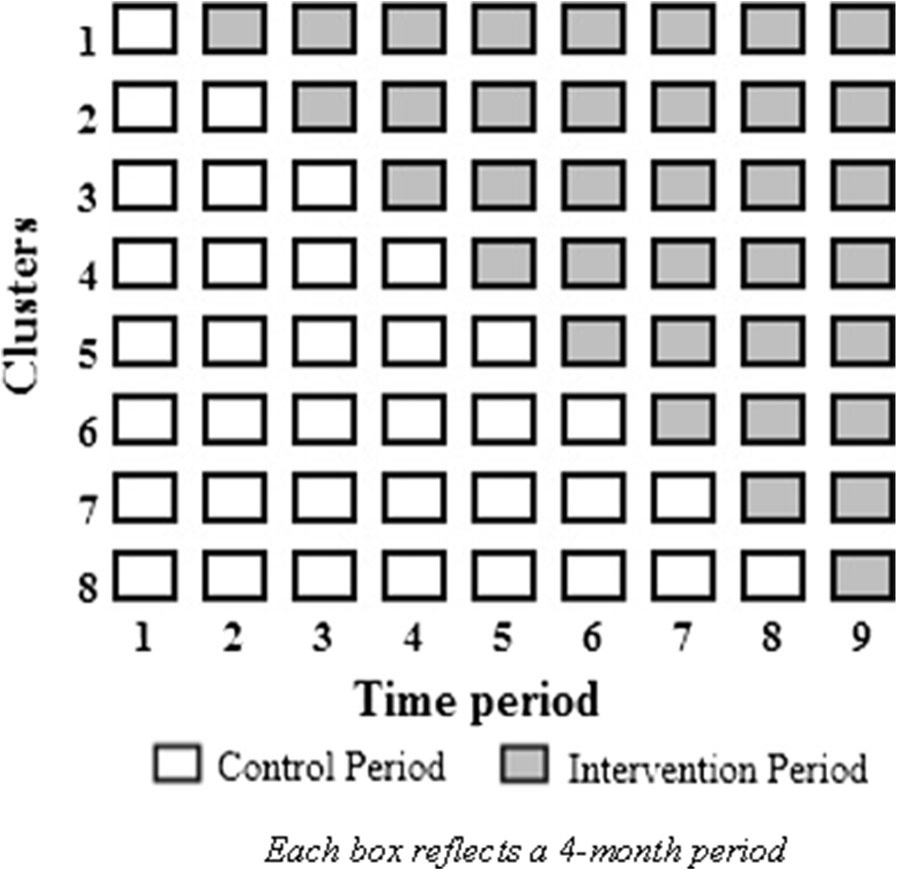
My Baby’s Movements stepped-wedge design. Stepped-wedged cluster design

This is a cluster-randomised, stepped-wedge design trial wherein maternity facilities are randomised in groupings (or clusters). All units will implement the MBM intervention at randomly-assigned points during the trial; these time points are the so-called “step” of the stepped-wedge design. The MBM trial design proposes sequential introduction of the intervention into eight groups of 3–5 hospitals at four-monthly intervals over a total of 3 years (Fig. 2).

Nested studies are planned including: cross sectional surveys to determine acceptability of information and knowledge on DFM; women’s use of MBM and perceptions of acceptability; clinical audits of presentations with DFM to determine changes in patterns of reporting of DFM and management; and focus groups studies to determine the acceptability of the intervention to women and clinicians.

### Study sites

Twenty-six maternity hospitals in Australia and New Zealand (ANZ).

### Study population

#### Inclusion criteria

Women with a singleton pregnancy attending for antenatal care; and midwives and doctors providing maternity care at the participating hospitals.

#### Exclusion criteria

Women with a lethal fetal congenital abnormality (CA).

Lethal fetal congenital abnormalities are defined as those that are unequivocally lethal.

### Sample size

The trial will include 26 hospitals in ANZ with an average of 3170 singleton births per year (range: 1400, 7000) giving 256,770 total births over 3 years. With a stillbirth rate 28 weeks’ or more gestation of 3 per 1000 we would expect (without the MBM intervention) 770 stillbirths (> 28 weeks), with 10% due to lethal congenital abnormalities where the intervention is unlikely to have an effect, leaving 693 stillbirths. MBM is hypothesised to reduce the rate to 2 per 1000, which is considered an achievable benchmark for a high income country and was the effect size observed in the Norwegian study [36]. We calculated statistical power using the methodology for stepped wedge designs proposed in Hussey and Hughes [57]. The calculation based on equations (#7) and (#8) assumes: significance level of 5%; analysis by generalised linear mixed model; births equally distributed across hospital groupings; baseline stillbirth rate 0.3%; intervention stillbirth rate 0.2%; an intra-class correlation (ICC) = 0.005 [58]. The ICC reflects the fact that for large clusters (*n* = 3170), the ICC is small. We propose sequential introduction of the intervention into eight groups of 3–5 hospitals at four-month intervals; over a total of 3 years (see Fig. 2). This will give 89% power to detect a 30% relative risk reduction in stillbirth rates (from 3/1000 to 2/1000), 85% power to detect a 25% reduction, and 80% power to for a 15% reduction. The trial methods have been harmonised with that of in the AFFIRM trial [39]. Combining data from the two trials, with an estimated 700,000 births, would give 89% power to detect a 10% decrease in stillbirth rates.

### Trial procedure

#### Randomisation and allocation

Clusters are assigned to the timing of the intervention (control and interventions periods) using a computer-generated random number table by the trial biostatistician (Michael Coory) who is not to be involved in the clinical aspects of the study. Randomisation is stratified by hospital size (< 3000 and > 3000 births/ year) and proximity to each other (groups of hospitals which are in close proximity to each other will be treated as strata).

#### Study group management

##### Control Period

Standard care: Standard care across these maternity services usually includes provision of the bi-national brochure to women [46] and management of women according to the recommended guidelines [24]. Key recommendations include that all pregnant women should receive information about what constitutes normal FM and advise that concerns for a decrease in movements should be reported to a health care provider without delay. Upon presentation for care, and exclusion of fetal death, recommended clinical care includes a cardiotocograph (CTG) to exclude imminent fetal demise followed by a thorough examination and testing for maternal fetal haemorrhage. In the presence of risk factors or concerns about fetal growth, an ultrasound scan should be performed. Specific recommendations on timing of birth are not provided.

##### Intervention Period

**Fig. 3:**
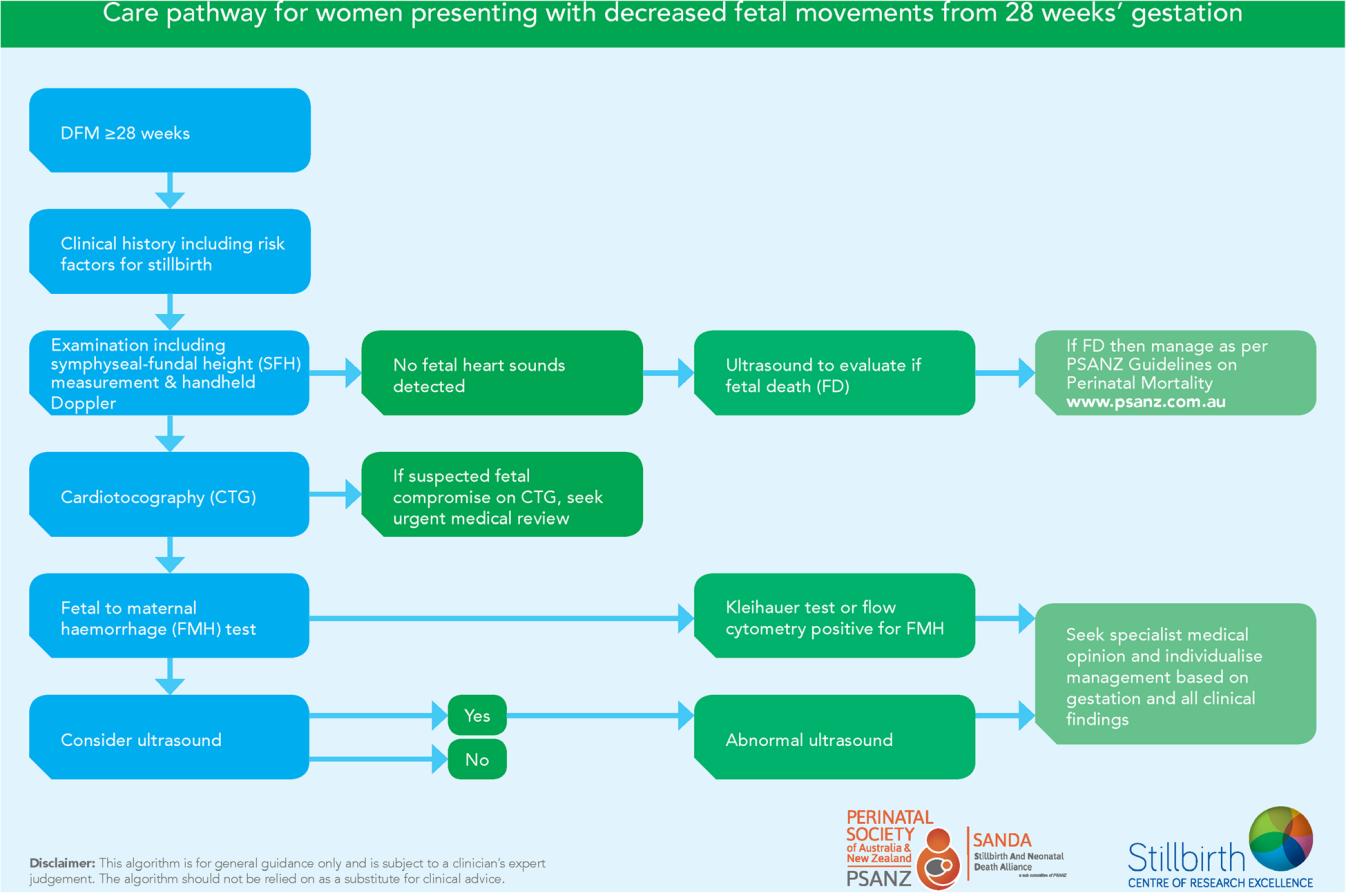
Care pathway for women presenting with decreased fetal movements from 28 weeks’ gestation. Clinical care management algorithm for women presenting with decreased fetal movements

MBM**:** In addition to standard care as provided in the control period, during the intervention period all eligible women will be offered the use of the MBM mobile phone app (or SMS messages for those who do not have a smartphone). All maternity care staff will be encouraged to complete an on-line DFM educational program, which educates and tests staff on the clinical care pathways for women presenting with decreased fetal movements, as outlined in Fig. 3. All sites receive a site visit from the CI team which includes presentation to staff on management of women with DFM.

Eight weeks prior to implementation, a teleconference is held with the MBM site team (usually made up of a midwifery educator, obstetrician and research midwife) and the MBM trial team to plan implementation, taking into consideration local procedures. The site team will be provided with an educational package about MBM and management of women with DFM to use in ongoing in-service education. One week prior to the commencement of the intervention phase, a site visit by the MBM trial team will be undertaken to present the trial and the management of women with DFM to clinical staff.

To access the MBM mobile phone program, each woman will be provided a unique Study ID generated through a purpose-built database. Registration will be undertaken by the staff at the maternity services. For women using the MBM app, the Study ID will be provided via SMS message on the day of registration or at 27 weeks’ gestation (whichever comes last). This message will include the woman’s unique MBM Study ID and instructions on how and where to download the MBM app. Once she has downloaded the app, the woman can sign-in to the app using her mobile phone number and unique MBM Study ID.

From 28 weeks’ gestation until birth the attending clinician will be asked to remind women about the use of MBM and reinforce the importance of being aware of DFM and when and how to contact the hospital. If the woman is found not to be currently registered in the MBM database, this can be arranged at any visit, regardless of gestation.

After the birth, women who have used the MBM app are given the option of completing a questionnaire embedded within the app. This is a 12-question survey asking women to rate app satisfaction, usefulness, and whether they would recommend MBM to others.

### Data collection

*Routinely collected electronic perinatal data*: will be accessed either through the health departments within each jurisdiction or hospitals. Data items are as follows:

i) Maternal demographics and obstetric history; previous stillbirth, previous miscarriage, previous neonatal death, previous FGR, previous preterm birth; maternal age; ethnicity; country of birth; body mass index; alcohol intake during pregnancy; smoking status at booking and at 20 weeks’ gestation; illicit drug use; education level; postcode; plurality; parity; Pre-existing major medical conditions including hypertension, diabetes, mental health and other.

ii) Pregnancy and birth outcomes; stillbirth, neonatal death, hypoxic ischemic encephalopathy, neonatal seizures; cause of neonatal death and stillbirth; gestation at birth; birthweight; FGR at birth; major congenital abnormality; Apgar Score < 7 at 5 min; umbilical artery pH < 7.0; intubation and ventilation at birth; Meconium Aspiration Syndrome; use of mechanical ventilation; neonatal death; reason for admission to nursery; onset of labour; mode of birth; major maternal pregnancy and birth complications including APH, pre-eclampsia, gestational hypertension, diabetes; maternal admission to intensive care; antenatal diagnosis of FGR.

Additional data items are as follows:

a) *Audit of presentations for DFM:* will be undertaken for two four-week periods prior to the commencement of the control period and at 6 months after the start of the intervention period using a purpose-built data collection form. Data collected will include the duration of maternal concern of DFM at the time of presentation, investigations undertaken and outcome of clinical assessment.

*b) Surveys of women:* will be undertaken over a four-week period immediately before commencement of the site education and again at 6 months after the start of the intervention period. Women will be asked during a routine antenatal visit at 35 weeks’ or more gestation or up to one-week post-partum to complete a survey to elicit psychosocial outcomes, knowledge and acceptability of the DFM information. A follow-up survey will be undertaken at 6 months postpartum by mail-out or by email (depending on the woman’s preference), to determine psychosocial outcomes, quality of life, and health services utilisation since discharge.

*c) Acceptability of the MBM Tool:* and factors that might inhibit utilisation will be assessed using qualitative methods. Four focus groups of 6–10 women, homogeneous for characteristics potentially associated with poorer uptake (young age; low socioeconomic status; Indigenous background) will be conducted towards the end of the intervention period. In addition, two focus groups of midwives and doctors will be undertaken towards the end of the intervention period. An experienced facilitator will use a semi-structured guide to elicit views and fresh insights into the intervention. Focus groups will be recorded and transcribed. Due to the impracticability of focus groups with multiple ethnic minority and other special needs groups, consultation and key informant interviews (e.g. with those who provide services for these specific population groups) will be conducted at participating hospital sites to gain insights into unique needs of specific populations served.

*d) Economic evaluation:* In addition to the routinely collected perinatal data and the health service use questionnaire to be completed at the 6 months follow-up time point, all Australian women completing the surveys as well as all women experiencing a stillbirth will be asked for consent to obtain their Medicare Benefits Schedule (MBS) and Pharmaceutical Benefits Scheme (PBS) claims data from the federal government via the Department of Human Services.

### Data management

Routinely collected perinatal data on singleton births over the three-year study period will be submitted electronically to the coordinating centre at the Mater Research Institute (MRI-UQ) by participating hospitals, or where hospitals do not have electronic data collection at the site, through the relevant health departments. Routine data will be provided in de-identified format, ensuring patient privacy and confidentiality. Where possible, data will be gathered electronically and entered directly into the purpose-built online database for the audit and surveys, in the case of paper format electronic scanning format will be utilised and entered by a member of the research team.

#### Data harmonisation for routinely collected data

From the 26 different facilities we expect 16 different system extracts. Due to the inconsistencies between systems, mapping will be undertaken to harmonise the datasets. Processes will include field mapping of similar fields and harmonisation of data points within these fields by mapping with the use of ICD-10 coding [59] and agreement by an expert panel consisting of investigators.

#### Linkage processes

Within the control period, datasets from women’s surveys and audits will be linked to birth outcomes via deterministic linkage processes [60]. This process will be completed via linking four common variables within each data set; maternal date of birth, estimated date of birth, hospital and timing of audit/survey. Within the intervention period, the research midwife at each site will enter re-identifiable data for eligible women into a purpose-built online database as follows: hospital record number, date of first antenatal visit, date of birth, and estimated date of confinement. The database will generate a unique MBM ID for each woman for use on audit forms and surveys. For women in the intervention period, data from the woman’s surveys, use of the MBM mobile phone program, and the DFM audits and birth outcomes will be linked using the MBM ID number.

### Analysis plan

#### Primary outcomes

To gain understanding of the population sample, initial analyses will involve examination of baseline characteristics of all women in the control and intervention periods, to provide an indication of comparability of the groups and identify potential confounders. Analyses of the primary outcome will be modelled upon analyses undertaken in the UK AFFIRM Trial [39]. To test the hypothesis that the MBM package results in a reduction in stillbirth rates at 28 weeks’ or more gestation, the binary primary outcome of stillbirth will be analysed via a generalised linear mixed model. This model will include a random effect for facility and fixed effects for the intervention implementation and study time periods. For further understanding please refer to the statistical analysis plan in the Additional file 1.

Intervention implementation (intention to treat group) will be determined by grouping women who were exposed to the intervention and those who were not, based upon the stepped wedge design. As there are multiple levels of intervention outlined within the study design, a further subset analysis will be undertaken utilising app data from women that utilised the MBM app. Utilisation of the MBM app will be determined as women who not only downloaded the app but accessed multiple pages of the app across multiple time periods. Baseline characteristics and similar analyses to the primary outcome analysis will be conducted for this group, along with time series analyses to understand specific app usage, stratified by gestation and demographical variables. Mixed model regression will be utilised to determine the differences in outcomes for women who received the MBM SMS program (non-smart phone users) and their birth outcomes.

#### Secondary Outcomes

Analysis of the secondary outcomes, will provide further understanding of the impact of the MBM package on birth and neonatal outcomes. Data will be analysed by generalised linear mixed models to identify the estimated adjusted odds ratio and 95% confidence interval for each of the birth outcomes and adverse neonatal outcomes identified in the aims. Outcomes measured on a continuous scale will be analysed in a normal linear mixed model. To determine the overall effectiveness of the intervention on secondary outcomes, analysis will involve comparison of the data points in the control section of the wedge with those in the intervention section, [61] adjusting for potential confounders, including maternal age, congenital abnormalities and gestational age etc.

#### Economic evaluation

The incremental cost effectiveness ratio (ICER) for the MBM intervention (i.e. the additional cost of an additional stillbirth avoided) will be calculated from trial data. Costs will include in-hospital and out-of-hospital service use (including scans and tests) and prescription medication use for the mother and baby. Hospital costs will be derived from Australian Refined Diagnosis Related Groups (AR-DRG) cost weights for any maternal or neonatal admission, out of hospital costs will be derived directly from the MBS and PBS data. The primary outcome of interest will be avoided stillbirth. A generalized linear mixed model will be utilised to compare total costs per birth in the intervention and control groups. The difference in the ICER between socioeconomic groups will also be compared. Additionally, maternal quality of life (QoL) will be measured using the AQoL8D [56] and health status using SF-36 [62]. The number of scans, caesarean sections, early inductions of labour and admissions to Neonatal Intensive Care Unit or Special Care Nursery will also be compared between intervention and control groups.

#### Qualitative data

Thematic analysis will be applied to the qualitative data collected throughout the study. Interviews will be recorded, or detailed notes will be taken at each qualitative data collection point. At least two researchers will read and independently establish coding categories before using an iterative approach to develop agreed key themes, with attention to any contrasts across groups. Stakeholder checks will be conducted where possible to allow participant groups and key informants to provide further comment on any resultant refinements made to the intervention.

#### Audit Data

Analysis of these datasets will compare data from the control and intervention periods, across hospitals providing baseline statistics of the two time periods. Descriptive and exploratory multivariate logistic regression analyses will be undertaken to understand health service utilisation across the different clusters. Audit data will be linked to birth outcomes, via above mentioned linkage processes for control and intervention data and will be analysed using the same methods as the secondary outcomes.

### MBM trial committees

A steering committee, made up of the trial chief investigators, will meet regularly to ensure successful completion of the trial. An independent data monitoring committee will make recommendations to the steering committee including early stopping due to safety concerns.

### Timeline and trial end

The trial will be undertaken over 5 years including 3 years of implementation of the interventions according to the randomisation schedule and data accrual. The control period will commence on the 8th of August 2016 and the last day of the intervention period is the 13th May 2019. Upon the trial end date, hospitals will provide their final routine data extract within 90 days to allow for a complete dataset.

## Discussion

Stillbirth is a common and devastating outcome with long lasting psychosocial impact for women and families. Many of these deaths are potentially avoidable. Maternal perception of DFM is a marker of an at-risk pregnancy and commonly precedes a stillbirth. However, suboptimal awareness by women of the importance of DFM and/or delay in seeking health care with concerns of DFM limits its potential. The delay is related to the lack of appreciation of the importance of FM as a result of inadequate information provided in busy maternity care settings. There is support in the community and in clinical practice of the need to ensure women receive better information and support about DFM during pregnancy. If effective, MBM offers a simple, inexpensive resource to reduce the numbers of stillborn babies and families suffering the distressing consequences of such a loss.

## Supplementary information

**Additional file 1.** Statistical Analysis Plan.

## Data Availability

The datasets used during the current study are available from the corresponding author upon reasonable request.

## Abbreviations

AFFIRM: Awareness of fetal movements and care package to reduce fetal mortality (AFFIRM): a stepped wedge, cluster-randomised trial
CA: Congenital abnormality
CTG: Cardiotocograph
DFM: Decreased Fetal Movements
EPDS: Edinburgh Postnatal Depression Scale
FGR: Fetal Growth Restriction
FM: Fetal movement
HIC: High Income Countries
ICC: Intra-class correlation
ICER: Incremental cost effectiveness ratio
IMPACT: Interdisciplinary Maternal and Perinatal Australasian Collaborative Trials
MBM: My Baby’s Movements
MBS: Medicare Benefits Schedule
MMH: Mater Mothers’ Hospital
PAI: Prenatal Attachment Inventory
PBS: Pharmaceutical Benefits Scheme
PSANZ: Perinatal Society of Australia and New Zealand
QoL: Quality of life
SMS: Short message service
