# Supplementary material for: My Baby’s Movements: a stepped wedge cluster randomised controlled trial to raise maternal awareness of fetal movements during pregnancy study protocol
Source: BMC Pregnancy Childbirth. 2019 Nov 21;19:430. doi: 10.1186/s12884-019-2575-1 (PMC6873438; doi:10.1186/s12884-019-2575-1)
Supplement: Supplementary file 1 — Additional file 1. Statistical Analysis Plan. [file 12884_2019_2575_MOESM1_ESM.docx]

My Baby’s Movements: a stepped wedge cluster randomised controlled trial to raise maternal awareness of fetal movements during pregnancy

Statistical Analysis Plan

**Authors:** Vicki Flenady, Kara Warrilow, Michael Coory, Megan Weller, Glenn Gardener, Philippa Middleton, David Ellwood, Katie Groom, Christine East, Emily Callander, Frances M Boyle, Aleena Wojcieszek, Adrienne Gordon, Jane Norman_,_ Caroline Crowther on behalf of the IMPACT Clinical Trials Network for Mothers’ and Babies’ Health.

**Trial registration:**  ACTRN12614000291684.

Registered 19 March 2014

**Based on Protocol Version**: 6.1, February 2018

**Chief Investigator:** Professor Vicki Flenady

**Co-Principle Investigator:** Glenn Gardener

**Trial Statistician:** Michael Coory

**Data Manager:** Kara Warrilow

**Trial Coordinator:** Megan Weller

| **Contact Details**  Centre of Research Excellence in Stillbirth  Mater Research  Level 3 Aubigny Place, Raymond Terrace  South Brisbane, 4101  Queensland, Australia | Professor Vicki Flenady  Tel: (+61) 07 3163 6349  Mobile: +61 419 664 956  Email: [vicki.flenady@mater.uq.edu.au](mailto:vicki.flenady@mater.uq.edu.au)  Email: [mbmtrial@mater.uq.edu.au](mailto:mbmtrial@mater.uq.edu.au)  Tel: (+61) 07 3163 6349 |
| --- | --- |

Contents

[1. Introduction 3](#_Toc10118223)

[Ethical Statement 3](#_Toc10118224)

[2. Study Design 3](#_Toc10118225)

[2.1 Brief Description 3](#_Toc10118226)

[2.2 Aims 3](#_Toc10118227)

[2.3 Hypothesis 3](#_Toc10118228)

[2.4 Inclusion Criteria 3](#_Toc10118229)

[3. Outcomes 4](#_Toc10118230)

[Outcome Measures 4](#_Toc10118231)

[3.1 Primary endpoint: 4](#_Toc10118232)

[3.2 Secondary endpoints are as follows: 4](#_Toc10118233)

[3.3 Subset and process outcomes: 4](#_Toc10118234)

[4. Statistical and Data management 5](#_Toc10118235)

[4.1 Data Collection 5](#_Toc10118236)

[4.2 Sample Size 5](#_Toc10118237)

[4.3 Randomisation 5](#_Toc10118238)

[4.4 Linkage 6](#_Toc10118239)

[4.5 Harmonisation 6](#_Toc10118240)

[5. General statistical considerations 6](#_Toc10118241)

[5.1 Handling of Missing data 7](#_Toc10118242)

[5.2 Demographic Variables 7](#_Toc10118243)

[6. Proposed outcome analyses 8](#_Toc10118244)

[6.1 Primary Outcome Analysis 8](#_Toc10118245)

[6.1.1Subset analyses of the primary outcome 8](#_Toc10118246)

[6.1.2 Sensitivity Analyses 8](#_Toc10118247)

[6.2 Secondary Outcomes: 9](#_Toc10118248)

[6.2.1 Discrete Secondary Outcomes 9](#_Toc10118249)

[6.4 Process Outcomes 10](#_Toc10118250)

[6.5 Exploratory analyses 10](#_Toc10118251)

[References 11](#_Toc10118252)

# Introduction

This document outlines the statistical analysis plan for the primary hypotheses analyses of the My Baby’s Movements trial. The document will provide details of variables, methodology and analyses undertaken. This document has been developed based upon information within the study protocol dated 2nd February 2018.

## Ethical Statement

Primary ethical approval was obtained from Mater Misericordiae Ltd Human Research Ethics Committee (EC00332) (MML HREC) in 2015. Further jurisdictional ethics approval was obtained from seven participating HRECs across Australia and New Zealand. Governance clearance was obtained for each of the 26 facilities involved in the trial. Amendments to the protocol and corresponding study documents will be provided for ethical and governance review prior to change. The control period of the study commenced on the 8th of August 2016. Recruitment for the intervention period began in November 2018, within the first cluster, and is due for completion in May 2019.

# Study Design

## 2.1 Brief Description

The My Baby’s Movements (MBM) Trial is a stepped-wedge, cluster randomised trial of maternal awareness and reporting of decreased fetal movements to reduce stillbirth led by the NHMRC Centre of Research Excellence in Stillbirth. The MBM package consists of a woman centred mobile phone application, which educates women and prompts timely reporting of DFM and an education program for clinicians around best-practice management of women reporting DFM.

The program has been rolled out to 26 maternity facilities across Australia and New Zealand over a period of 3 years. The trial acts in an intention to treat manner and will aim to identify if whether increased awareness of DFM [offering the MBM package to hospitals] can will decrease stillbirth rates.

## 2.2 Aims

The aim of the MBM trial is to evaluate the effectiveness of a mobile phone application for women combined with an educational program for clinicians (MBM intervention/package) in reducing stillbirth rates for gestations ≥ 28 weeks.

## 2.3 Hypothesis

The primary hypothesis is that the MBM intervention/package will result in a reduction in stillbirth rates at 28 weeks’ or more gestation in women with a singleton pregnancy from 3/1000 to 2/1000. The baseline stillbirth rate of 3/1000 is the current aggregate stillbirth rate at the participating hospitals.

## 2.4 Inclusion Criteria

***Inclusion criteria:*** Women with a singleton pregnancy attending for antenatal care; and midwives and doctors providing maternity care at the participating hospitals.

***Exclusion criteria:*** Women with a lethal fetal congenital abnormality (CA).

# Outcomes

## Outcome Measures

### 3.1 Primary endpoint:

Stillbirth rates 28 weeks’ or more gestation in women with a singleton pregnancy

### 3.2 Secondary endpoints are as follows:

1. Key secondary endpoints:

- Rate of Caesarean Section (CS)
- Rate of Admission to Neonatal Intensive Care Unit (NICU)
- Rate of Induction of Labour (IOL)

1. Exploratory secondary endpoints

- Stillbirth rate by gestation - stratified by weeks;
- Fetal Growth Restriction rate
- Obstetric outcomes: intrapartum and postpartum infection; postpartum haemorrhage; maternal admission to intensive care.
- Composite adverse newborn outcome score defined as one or more of the following in a livebirth born at ≥28 weeks gestation [1, 2]:
- neonatal death (death of a live born infant regardless of birthweight)
- Apgar Score <7 at 5 minutes
- hypoxic ischemic encephalopathy (neonatal)
- neonatal seizures
- Meconium Aspiration Syndrome
- umbilical artery pH <7.0
- intubation and ventilation at birth
- NICU admission >48 hours

### 3.3 Subset and process outcomes:

Data for the following analyses will be utilised from audit and survey data undertaken during recruitment of the MBM trial. Sample size for the following sub sets is expected to be <8000.

1. Health service utilisation measures: episodes of women presenting with DFM at ≥28 weeks’ gestation; antenatal admission to hospital for DFM; antenatal ultrasound; duration of neonatal intensive care, special care nursery and total hospital stay; and maternal length of hospital stay.
2. Woman’s psychosocial outcomes and health seeking behaviour and acceptability: Maternal reporting of DFM delayed by > 24 hours; acceptability of information on DFM and of MBM; women’s and clinicians’ knowledge of FM; maternal-fetal attachment (the Prenatal Attachment Inventory (PAI);[3] maternal pregnancy-related worries and concerns (the Cambridge Worry Scale Score;[4]) anxiety (State-Trait Anxiety Index[5]); the Edinburgh Postnatal Depression Scale (EPDS);[6] quality of life (QoL)(AQol8D);[7] and health status (SF36)[8] at the end of pregnancy (or birth) at 6 months postpartum.

# Statistical and Data management

## 4.1 Data Collection

Data will be obtained from all participating hospital via electronic perinatal records. This will be accessed either through the individual hospitals or through jurisdictional health departments, dependant on hospital preference.

Data will be collected on an annual basis for the three years and provided to the coordinating centre via a password encrypted file transfer. All data is to be sent to the coordinating centre de-identified and will be stored for management and analysis under password encrypted filing with worksheets accessible only to the study investigators. Hospitals have a responsibility to ensure that there are no names, addresses or URN’s in all extracts provided to the coordinating centre. All variables, including potentially identifiable variables such as postcode and birth date have been approved by all Australian and New Zealand Ethics.

## 4.2 Sample Size

The trial will include 26 hospitals in ANZ with an average of 3,170 singleton births, of at least ≥ 28 weeks gestation, per year (range: 1400, 7000) giving 256,770 total births over three years. With a stillbirth rate 28weeks’ or more gestation of 3 per 1000 we would expect (without the MBM intervention) 770 stillbirths (>28 weeks), with 10% due to lethal congenital abnormalities where the intervention is unlikely to have an effect, leaving 693 stillbirths. The MBM intervention is hypothesised to reduce the rate to 2 per 1000, which considered an achievable benchmark for a high income country and was the effect size observed in the Norwegian study.[9] We calculated statistical power using the methodology for stepped wedge designs proposed in Hussey and Hughes.[10] The calculation based on equations (#7) and (#8) assumes: significance level of 5%; analysis by generalised linear mixed model; births equally distributed across hospital groupings; baseline stillbirth rate 3/1000; intervention stillbirth rate 2/1000; an intra-class correlation (ICC)=0.005.[11] The ICC reflects the fact that for large clusters (n=3170), the ICC is small. We propose sequential introduction of the intervention into eight groups of 3-4 hospitals at four-month intervals; over a total of three years (see Figure 2). This will give 89% power to detect a 30% relative risk reduction in stillbirth rates (from 3/1000 to 2/1000), 85% power to detect a 25% reduction, and 80% power to for a 15% reduction. The trial methods have been harmonised with that of in the AFFIRM trial.[12] Combining data from the two trials, with an estimated 700,000 births, would give 89% power to detect a 10% decrease in stillbirth rates.

## 4.3 Randomisation

Clusters are assigned to the timing of the intervention (control and interventions periods) using a computer-generated random number table by the trial biostatistician (Michael Coory) who will not be involved in the clinical aspects of the study. Randomisation is stratified by hospital size (<3000 and >3000 births/ year) and proximity to each other (groups of hospitals which are in close proximity to each other will be treated as strata).

## 4.4 Linkage

Within the control period, datasets from women’s surveys and audits will be linked to birth outcomes via four common variables within each data set; maternal date of birth, estimated baby date of birth, hospital and timing of audit/survey. Within the intervention period, the research midwife at each site will enter re-identifiable data for eligible women into a purpose-built on-line database as follows: hospital record number, date of first antenatal visit, date of birth, and estimated date of confinement. The database will generate a unique MBM ID for each woman for use on audit forms and questionnaires. For women in the intervention period, data from the woman’s surveys, use of the MBM mobile phone program, DFM audits and birth outcomes will be linked using the MBM ID number.

## 4.5 Harmonisation

From the 26 different facilities we expect 16 different system extracts. Due to the inconsistencies across systems, mapping will be undertaken to harmonise the datasets. Processes will include field mapping of similar fields and harmonisation of data points within these fields by mapping with the use of ICD-10 coding [13] and agreement by an expert panel consisting of chief investigators.

# General statistical considerations

Statistical analyses and data management will be conducted by the trial statistician and data manager using the statistical packages STATA V15[14] and R 3.6.0[15], dependant on analyses and modelling conducted. Analyses of the primary outcome/endpoint will be aligned with the analyses undertaken in the UK AFFIRM Trial. [12]

To gain understanding of the population sample, initial analyses will involve examination of baseline characteristics of all women in the control and intervention periods, to provide an indication of the baseline characteristics (underlying risk factors for stillbirth) of the groups and identify any changes in the underlying risk factors for stillbirth over time. Categorical data will be summarised using percentages and counts; continuous variables will be summarised using means, standard deviations, medians, interquartile ranges, and minimum and maximum values, as appropriate.

All analyses for primary and secondary outcomes will follow the Intention to Treat principle (ITT), with births analysed according to whether they took place during the control or intervention periods, irrespective of whether the intervention had been implemented as planned. Secondary on-treatment analyses will assign a birth to the control period if a site was non-adherent to the MBM intervention at the time of the birth. The primary outcome/endpoint (stillbirths ≥ 28 weeks gestation) will be analysed by a cluster-period mixed-effects model, with a logit link, to estimate the adjusted OR (aOR) and 95% CI for the intervention period versus the control period. Pre-specified potential confounders that will be included in the model are: maternal age, maternal BMI at booking, smoking after 20 weeks gestation, country of birth, indigenous status, major pregnancy complications (antepartum haemorrhage, pre-eclampsia, gestational diabetes) and major medical conditions (including diabetes, renal disease).

Adjusted absolute and relative risk differences will be calculated to aid communication of the results.

The primary hypothesis (a decrease in stillbirth rates) will be assessed using a two-sided p-value for the adjusted odds ratio as per the sample size calculation; p<0.05 (two-sided) (95% confidence interval will also be provided). For the secondary endpoints, the p-values and confidence intervals will be nominal (i.e., the secondary endpoint analyses will not be adjusted for statistical multiplicity).

## 5.1 Handling of Missing data

No imputation or will be performed for missing data regarding withdrawals or missing values in analyses and summaries. Missing data from variables not collected by jurisdictions will be left missing within analyses, unless otherwise stated.

Where Ethnicity is missing, ethnic origin will be inferred from Country of Birth. Where reporting of DFM is missing, but Reason for Induction of Labour (IOL) states DFM, data will be imputed.

## 5.2 Demographic Variables

Demographics will be analysed in an exploratory baseline manner. These will be reported in table form and will identify comparability groups and potential confounding variables.

**Maternal Characteristics:**

- Maternal Age at birth of baby (Years)
- Maternal Ethnicity
- Maternal Indigenous Status
- Maternal Country of Birth
- BMI at Booking
- Maternal height (cm)
- Maternal weight at booking (kg)
- Postcode
- Parity
- Plurality
- Alcohol intake in pregnancy
- Smoking status during pregnancy
- Smoking history at booking
- Edinburgh Depression Scale score at birth

**Baby Characteristics:**

- Baby’s Birthweight
- Gestation at birth
- Baby Gender
- Apgar score at birth, 5 minutes and 10 minutes

# 6. Proposed outcome analyses

## 6.1 Primary Outcome Analysis

Analyses of the primary outcome will be similar to the analyses undertaken in the UK AFFIRM Trial.[12] To test the hypothesis that the MBM package results in a reduction in stillbirth rates at 28 weeks or more gestation, the binary primary outcome of stillbirth will be analysed via a cluster-period mixed-effects model, with a logit link.[16-18]

$$logit(P(Y_{ijk}-1)=\mu+\beta_{j}+\theta X_{ij}+u_{i}+q_{ij}$$

where

Y*_ijk_* denotes whether a stillbirth occurred for pregnancy-k, for period/time-j, at hospital-i

B*_j_* is the change in stillbirth rate (if any) from the first period to period/time-j

Ɵ is the effect of the My-Baby’s-Moments intervention

X*_ij_* is a binary variable denoting exposure to the My-Baby’s-Moments intervention

u*_i_* ~ N(0, σ2) is the random effect for each cluster

q*_ij_* ~ N(0, σ2) is the random effect for each period

Analyses will also be done using non-parametric with-period methods to confirm the robustness of the results. [19]

## 6.1.1Subset analyses of the primary outcome

1. MBM App usage

Secondary analyses of the primary endpoint (stillbirths) will involve the multiple levels of intervention outlined within the study design. A subset analysis will be undertaken utilising app data from women that utilised the MBM app. Utilisation of the MBM app will be determined as women who not only downloaded the app but accessed multiple pages of the app across multiple time periods. Baseline characteristics and similar analyses to the primary outcome analysis will be conducted for this group, along with time series analyses to understand specific app usage, stratified by gestation and demographical variables. Mixed model regression will be utilised to determine the differences in outcomes for women who received the MBM SMS program (non-smart phone users) and their birth outcomes.

1. Primiparity by multiparity.

A subset analysis of stillbirths will be undertaken utilising methods outlined in section 6.1 for nulliparas and multiparas’ women. P-values and CIs will be nominal.

## 6.1.2 Sensitivity Analyses

We will explore the analyses over a time series analysis to monitor effects that may have occurred across the period which may have impacted the results of the primary and secondary outcomes. This will be completed by modelling the intervention periods by time using nonlinear nonparametric analyses.

- Maternal Age
- Maternal BMI at booking
- Smoking after 20 weeks gestation
- Country of birth
- Indigenous status
- Major pregnancy complications (antepartum haemorrhage, pre-eclampsia, gestational diabetes)
- Major medical conditions (including diabetes, renal disease)

## 6.2 Secondary Outcomes:

Analysis of the secondary outcomes will provide further understanding of the impact of the MBM package on birth and neonatal outcomes. Data will be analysed by generalised linear mixed models to identify the estimated adjusted odds ratio and 95% confidence interval for each of the birth outcomes and adverse neonatal outcomes identified in the aims. Outcomes measured on a continuous scale will be analysed in a normal linear mixed model. To determine the overall effectiveness of the intervention on secondary outcomes, analysis will involve comparison of the data points in the control section of the wedge with those in the intervention section,[20] adjusting for potential confounders, including maternal age, congenital abnormalities and gestational age etc.

### 6.2.1 Discrete Secondary Outcomes

The following outcomes will be analysed in methods outlined for the primary outcome in section 6.1.

- Rate of neonatal death
- Rates of caesarean section
- Rates of induction of labour
- Rates of spontaneous vaginal birth
- Rates of admission to Neonatal intensive care unit (NICU)
- Rates of admission to NICU >48 hours
- Rates of admission to Special Care Nursery (SCN)
- Proportion of preterm infants
- Rate of infants born with an Apgar Score <7
- Rate of babies requiring intubation or ventilation at birth
- Rates of babies treated for neonatal seizures
- Rates of babies treated for hypoxic ischemic encephalopathy (HIE)
- Rate of babies with confirmed Fetal Growth Restriction (FGR)

6.3 Sub-study Analyses

a) Economic evaluation:

The incremental cost effectiveness ratio (ICER) for the MBM intervention (i.e. the additional cost of an additional stillbirth avoided) will be calculated from trial data. Costs will include in-hospital and out-of-hospital service use (including scans and tests) and prescription medication use) for the mother and baby. Hospital costs will be derived from Australian Refined Diagnosis Related Groups (AR-DRG) cost weights for any maternal or neonatal admission, out of hospital costs will be derived directly from the MBS and PBS data. The primary outcome of interest will be avoided stillbirth. A generalized linear mixed model will be utilised to compare total costs per birth in the intervention and control groups. The difference in the ICER between socioeconomic groups will also be compared. Additionally, maternal quality of life (QoL) will be measured using the AQoL8D[7] and health status using SF-36.[8] The number of scans, caesarean sections, early inductions of labour and admissions to Neonatal Intensive Care Unit or Special Care Nursery will also be compared between intervention and control groups.

b) Qualitative data:

Thematic analysis will be applied to the qualitative data collected throughout the study. Interviews will be recorded, or detailed notes will be taken at each qualitative data collection point. At least two researchers will read and independently establish coding categories before using an iterative approach to develop agreed key themes, with attention to any contrasts across groups. Stakeholder checks will be conducted where possible to allow participant groups and key informants to provide further comment on any resultant refinements made to the intervention.

c) Audit Data:

Analysis of these datasets will compare data form the control and intervention periods, across hospitals providing baseline statistics of the two time periods. Descriptive and exploratory multivariate logistic regression. analyses will be undertaken to understand Health service utilisation across the different clusters. Audit data will be linked to birth outcomes, via above mentioned linkage processes for control and intervention data and will be analysed using the same methods as the secondary outcomes.

## 6.4 Process Outcomes

The following research outcomes will be identified from the facility audits completed pre and post intervention.

**Discrete**

- Number of women presenting with decreased fetal movements
- Number of IOL interventions performed upon presentation for decreased fetal movements

**Continuous**

- Time of woman first perceiving reduced fetal movements and presentation to hospital
- Time of last movement felt by woman and presentation

## 6.5 Exploratory analyses

The following research questions will be answered in subset analyses undertaken using linked routine data to facility audits and from surveys undertaken in late pregnancy.

- Change in stillbirths by investigations undertaken upon presentation for decreased fetal movements.
- Investigations performed in comparison to best practice clinical guidelines upon presentation of decreased fetal movements
- Women’s perception of decreased fetal movements in late pregnancy and how information was provided by health care providers

## References

1. Grobman WA, Rice MM, Reddy UM, Tita ATN, Silver RM, Mallett G, et al. Labor Induction versus Expectant Management in Low-Risk Nulliparous Women. New England Journal of Medicine. 2018;379(6):513-23. doi: 10.1056/NEJMoa1800566.

2. Chow SW CP, Kander V, Haslam R, Lui Kei. Report of the Australian and New Zeasland Neonatal Network 2016. Sydney 2016.

3. Muller ME. Development of the Prenatal Attachment Inventory. West J Nurs Res. 1993;15(2):199-211; discussion -5. Epub 1993/04/01. PubMed PMID: 8470375.

4. Statham H, Green JM. The effects of miscarriage and other ‘unsuccessful’ pregnancies on feelings early in a subsequent pregnancy. J Reprod Infant Psychol. 1994;12(1):45-54. doi: 10.1080/02646839408408867.

5. Spielberger CD, Gorsuch RL, Lushene R, Vagg PR, Jacobs GA. Manual for the State-Trait Anxiety Inventory. Palo Alto, CA: Consulting Psychologists Press; 1983.

6. Cox JL, Holden JM, Sagovsky R. Detection of postnatal depression. Development of the 10-item Edinburgh Postnatal Depression Scale. Br J Psychiatry. 1987;150(6):782-6. doi: 10.1192/bjp.150.6.782.

7. Richardson J, Iezzi A. Psychometric validity and the AQoL-8D multi attribute utility instrument. Melbourne: Centre for Health Economics, Monash University; 2011.

8. Brazier JE, Harper R, Jones NMB, Ocathain A, Thomas KJ, Usherwood T, et al. Validating the SF-36 health survey questionnaire - new outcome measure for primary care. Brit Med J. 1992;305(6846):160-4. PubMed PMID: ISI:A1992JF06300028.

9. Tveit JV, Saastad E, Stray-Pedersen B, Bordahl PE, Flenady V, Fretts R, et al. Reduction of late stillbirth with the introduction of fetal movement information and guidelines - a clinical quality improvement. BMC Pregnancy Childbirth. 2009;9:32. Epub 2009/07/25. doi: 1471-2393-9-32 [pii]

10.1186/1471-2393-9-32 [doi]. PubMed PMID: 19624847; PubMed Central PMCID: PMC2734741.

10. Hussey MA, Hughes JP. Design and analysis of stepped wedge cluster randomized trials. Contemp Clin Trials. 2007;28(2):182-91. doi: <http://dx.doi.org/10.1016/j.cct.2006.05.007>.

11. Campbell MJ, Donner A, Klar N. Developments in cluster randomized trials and Statistics in Medicine. Statistics in Medicine. 2007;26(1):2-19. doi: 10.1002/sim.2731.

12. Norman JE, Heazell AEP, Rodriguez A, Weir CJ, Stock SJE, Calderwood CJ, et al. Awareness of fetal movements and care package to reduce fetal mortality (AFFIRM): a stepped wedge, cluster-randomised trial. The Lancet. 2018;392(10158):1629-38. doi: 10.1016/S0140-6736(18)31543-5.

13. World Health Organization (WHO). International Statistical Classification of Diseases and Related Health Problems 10th Revision. Geneva: World Health Organization, 2016.

14. StataCorp. Stata Statistical Software: Release 15. College Station, TX: StataCorp LLC; 2017.

15. R Development Core Team (2008). R: A language and environment for

statistical computing. R Foundation for Statistical Computing,. Vienna, Austria.

16. Hooper R, Teerenstra S, Hoop E, Eldridge S. Sample size calculation for stepped wedge and other longitudinal cluster randomised trials. Statistics in Medicine. 2016;35(26):4718-28. doi: 10.1002/sim.7028.

17. Girling AJ, Hemming K. Statistical efficiency and optimal design for stepped cluster studies under linear mixed effects models. Statistics in Medicine. 2016;35(13):2149-66. doi: 10.1002/sim.6850.

18. Hemming K, Taljaard M, Forbes A. Analysis of cluster randomised stepped wedge trials with repeated cross-sectional samples.(Report). Trials. 2017;18(1). doi: 10.1186/s13063-017-1833-7.

19. Thompson JA, Davey C, Fielding K, Hargreaves JR, Hayes RJ. Robust analysis of stepped wedge trials using cluster‐level summaries within periods. Statistics in Medicine. 2018;37(16):2487-500. doi: 10.1002/sim.7668.

20. Brown CA, Lilford RJ. The stepped wedge trial design: a systematic review. BMC Med Res Methodol. 2006;6:54. Epub 2006/11/10. doi: 1471-2288-6-54 [pii]

10.1186/1471-2288-6-54. PubMed PMID: 17092344; PubMed Central PMCID: PMC1636652.
